# Downregulation of UBC9 promotes apoptosis of activated human LX-2 hepatic stellate cells by suppressing the canonical NF-κB signaling pathway

**DOI:** 10.1371/journal.pone.0174374

**Published:** 2017-03-30

**Authors:** Sufen Fang, Jinhua Yuan, Qing Shi, Tiantian Xu, Yao Fu, Zheng Wu, Wuhua Guo

**Affiliations:** 1 Department of Gastroenterology, Second Affiliated Hospital of Nanchang University, Nanchang, China; 2 Department of interventional radiology, Mengchao Hepatobiliary Hospital of Fujian Medical University, Fuzhou, China; Saint Louis University, UNITED STATES

## Abstract

UBC9, the only known E2-conjugating enzyme involved in SUMOylation, is a key regulator in fibrosis. However, the roles of UBC9 in liver fibrosis remain unclear. Therefore, in this study, we investigated the roles of UBC9 in HSC apoptosis and liver fibrogenesis. Our results showed that the UBC9 levels in activated LX-2 cells, HepG2 and SMMC-7721 were increased compared with LO2, and the expression of UBC9 in activated LX-2 cells, HepG2 and SMMC-7721 were no significant differences. The expression of UBC9 was effectively down-regulated by the UBC9-shRNA plasmid, and this effect was accompanied by the attenuated expression of the myofibroblast markers smooth muscle actin (α-SMA) and Collagen I. Downregulation of UBC9 also promotes activated HSCs apoptosis by up-regulating cell apoptosis-related proteins. Further, knockdown of UBC9 in activated HSCs inhibited cell viability and caused cell cycle arrest in the G2 phase. Moreover, knockdown of UBC9 suppressed the activation of NF-κB signaling pathways. In conclusion, these results demonstrated that down-regulation of UBC9 expression induced activated LX-2 cell apoptosis and promoted cells to return to a quiescent state by inhibiting the NF-κB signaling pathway. These results provide novel mechanistic insights for the anti-fibrotic effect of UBC9.

## Introduction

Hepatic fibrosis is an integral component in the progression of chronic inflammatory liver disease, which features excessive accumulation of extracellular matrix (ECM) proteins. With prolonged liver damage, fibrosis may progress to cirrhosis and primary liver cancer [1]. Unlike irreversible cirrhosis, hepatic fibrosis is a reversible disease, and an effective treatment can prevent or reverse the fibrotic process [2]. Hepatic stellate cells (HSCs) play a key role in liver fibrogenesis [3]. HSCs are quiescent in the normal liver but are activated in response to liver damage [4]. After activation, HSCs are converted to myofibroblasts, a rich source of Collagen I and a-SMA, which are proliferative, fibrotic and contractile. Activated HSCs secrete several factors, including transforming growth factor β (TGF-β), platelet-derived growth factor (PDGF) and other factors that promote the development and progression of liver fibrosis[5]. In addition, these activated HSCs also secrete tumor necrosis factor α (TNF-α), IL-6, human growth factor (HGF), fibroblast growth factor (FGF) and other cytokines[6]. This network of autocrine and paracrine cytokines regulates the development and progression of hepatic fibrosis. Therefore, restraining HSC activation and promoting HSC apoptosis are important measures for the prevention and treatment of liver fibrosis.

SUMOylation is a post-translational modification mediated by Small Ubiquitin-like Modifier (SUMO). This process controls a diverse array of cellular functions, such as the cell cycle, apoptosis, signal transduction pathways [7–9], production of reactive oxygen species and the inflammatory response [10]. UBC9 is the only known E2-conjugating enzyme involved in SUMOylation [11]. Therefore, UBC9 is a key regulator of fibrosis through SUMOylation. For example, knockdown of UBC9 prevents bleomycin-induced fibrosis[12]. Scholars have also demonstrated that inhibition of SUMOylation by knockdown of UBC9 almost completely prevented the development of fibrosis and inhibited the canonical TGF-β/Smad signaling pathway in the pathogenesis of SSc [13]. Therefore, we hypothesized that UBC9 may play a critical role in the occurrence and development of liver fibrosis.

The transcription factor nuclear factor-kappa B (NF-κB) is essential for liver cell survival and liver homeostasis[14]. Regulation of cell death, inflammation, and wound healing by NF-κB not only emphasizes the role of this transcription factor in the progression of liver diseases but also highlights the mechanistic links among liver injury, inflammation, fibrosis, and hepatocellular carcinoma[15]. Several studies have indicated that NF-κB inhibition is a potential mechanism for the induction of HSC apoptosis[16,17]. Hence, when NF-κB activation is prevented or inhibited, apoptosis of activated HSCs is enhanced.

Interestingly, a growing body of evidence has emphasized a potential role for UBC9 in organ fibrosis. For example, knockdown of UBC9 prevents bleomycin-induced fibrosis[12]. In addition, SUMO-1 and UBC9 overexpression decreases NOS2 (iNOS) promoter activity and suppresses the proinflammatory response in astrocytes[13]. To date, the mechanism of UBC9 in hepatic fibrosis remains unknown. In this study, these results demonstrated that down-regulation of UBC9 expression induced activated LX-2 cell apoptosis and promoted cells to return to a quiescent state by inhibiting the NF-κB signaling pathway.

## Materials and methods

### Cell culture

Two human hepatocellular carcinoma (HCC) cell lines, HepG2 and SMMC-7721, and liver cell lines, LO2 and LX-2, were obtained from the China Center for Type Culture Collection (CCTC, China) and cultured in a humidified incubator at 37°C with 5% CO_2_. HepG2 was cultured in minimum essential medium (DMEM, Gibco, USA). SMMC-7721, L02 and LX-2 were cultured in RPMI-1640 (Gibco, USA). The culture media described above were supplemented with 10% fetal bovine serum (FBS, Gibco, USA) 100 U/mL penicillin and 100 mg/mL streptomycin.

### Transfection

The cDNA sequence of UBC9 was obtained from GenBank. UBC9 shRNA: F, TGC TGT TAT GAG GGC GCA AAC TTC TTG TTT TGG CCA CGA CTG AC; R, CCT GTT ATG AGG GCG AAC TTC TTG TCA GTC AGT GGC CAA AAC AA, Negative control: F, tgctg AAA TGT ACT GCG CGT GGA GAC GTT TTG GCC ACT GAC TGA CGT CTC; R, cctg AAA TGT ACT GCG TGG AGA CGT CAG TCA GTG GCC AAA ACG TCT CCA. Both plasmids, which were fluorescently labeled, were provided by Invitrogen (Shanghai, China). The transfection of UBC9 shRNA was performed with Lipofectamine 2000 (Invitrogen, Shanghai, China) according to the manufacturer’s instructions. Untreated activated LX-2 cells served as the control (non-treated cells).

### Semi-quantitative RT–PCR

Total RNAs were extracted with Trizol (Invitrogen, Shanghai, China) from activated LX-2 cells. The concentration of RNA was determined by absorption measurements at 260 nm using a UV–visible spectrophotometer (Bio-Rad, USA). The reaction mixture was incubated for 10 min at 70°C and cooled rapidly on ice water for 5 min. Next, 1 μl of M-MLV reverse transcriptase (Promega, Shanghai, China), 1 μl of Rnase inhibitor (Promega, Shanghai, China), 1 μl of dNTP (Generay Biotech, China), 4 μl of M-MLV RT 59 buffer (Promega, Shanghai, China), and Rnase-free water were added to each reaction mixture for a final volume of 20 μl. The reaction mixture was incubated at room temperature for 10 min at RT followed by 42°C for 60 min. Finally, the mixture was incubated at 95°C for 5 min to terminate the reaction. For amplification of specific cDNAs, oligomer primers were designed for the following genes: UBC9 (Sense: 5-CAG GAA AGA AAG GGA CTC-3; Antisense: 5-TTC GGG TGA AAT AAT GG-3), β-actin: (Sense: 5-GCA TCC TGC ACC ACC AAC T-3; Antisense: 5-GCA GTG ATG GCA TGG ACT GT-3). The above primers were synthesized by Invitrogen (Shanghai, China). PCR was performed in a reaction mixture containing 1 μl of sense primer, 1 μl of antisense primer, 500 ng of cDNA, 12.5 μl of 2×Reaction Mix (Tiangen, China) and ddH_2_O, giving a final volume of 25 μl. Thirty cycles of denaturation (30 s at 94°C), annealing (30 s, at 55°C for UBC9 and β-actin), and extension (30 s at 72°C) were performed followed by a final extension at 72°C for 7 min in a PCR thermal cycler (GeneAmp@ PCR System 9600). PCR products were electrophoresed on a 2.5% agarose gel containing ethidium bromide and visualized with UV light. Quantification of band intensity was performed using the GeneGenius Match system (Syngene, USA).

### Western blot analysis

Total protein was extracted from cell pellets. The protein concentrations were determined using the BCA protein assay (Thermo Fisher Scientific, Rockford, USA). Then, thirty micrograms of each sample was separated by 10% SDS–polyacrylamide gel electrophoresis and transferred to a PVDF membrane (0.22 micrometer, Bio-Rad). The membrane was then blocked with 5% skim milk at room temperature. Rabbit polyclonal anti-α-SMA, Collagen I, p65, P-p65 and UBC9 (Abcam, USA)) were diluted 1:1000. Bax and Bcl-2 (Proteintech, Wuhan, China) were diluted 1:200. Rabbit monoclonal antibodies against Caspase 3 and cleaved-Caspase3 (cell Signaling, USA) were diluted 1:1000, and a mouse monoclonal antibody directed against β-actin (Proteintech, Wuhan, China) was used at 1:1000. The membrane was incubated with these antibodies overnight at 4°C. The membranes were washed three times with TBS/Tween 20 (0.075%) containing 3% Marvel for 15 min each before incubation with HRP-conjugated secondary antibodies (1:10,000) at 37°C for 1 h. The PVDF membrane was washed in TBST three times (10 min each time). The proteins were visualized using the Amersham^™^ ECL Plus Western Blotting Detection System (GE Healthcare, UK).

### MTT assay

To assess cell viability following treatment with an efficient transfection with the UBC9 shRNA duplex, 5×10^3^ activated LX-2 cells were plated in each well of a 96-well plate and treated with UBC9 shRNA. The cells were treated with a negative control or Lipofectamine alone, and non-treated cells served as control groups. The cells were treated in triplicate for each group. After incubation for 12 h at 37°C, cells were transfected according to the manufacturer’s instructions. Following 24 h, 48 h, and 72 h treatment with various reagents, 20 ml of a MTT (Sigma,USA) solution (5.0 mg/ml) was added to each well, and the cells were incubated again for 4 h in a humidified incubator at 37°C with 5% CO_2._ Then, 200 μl of a dimethylformamide solution was added to each well, and cells were incubated for 20 min at 37°C with 5% CO_2._ The absorbance at 450 nm was measured by an ELISA plate reader (DENLEY DRAGON MK2).

### Flow cytometry assay

Cells were seeded at a density of 5 × 10^5^ cells per well in 6-well plates. After treatment with UBC9 shRNA, the cells were trypsinized and collected by centrifugation. After washing twice with PBS and fixing in ice cold 70% ethanol, the cell cycle distribution was analyzed using the Cell Cycle Analysis Kit (MultiSciences, China), and apoptosis was detected using the Annexin V/PI Apoptosis Detection Kit I (BD BioSciences, USA) according to the manufacturer’s instruction. Data were collected and analyzed with BD FACSCalibur System.

### ELSA assay

Activated LX-2 cells were incubated in 6 cm plates for 12 h at 37°C with 5% CO_2_ before transfection of the UBC9 shRNA duplex according to the manufacturer’s instructions. Cells transfected with the negative control or treated with Lipofectamine alone served as controls. After incubation for 24 h, supernatants were collected by centrifugation. Then, 100 μL dilutions of standard, blanks and samples were added into the appropriate wells and incubated for 2 hours at 37°C. After removing the liquid from each well, 100 μL of Detection Reagent A working solution was added to each well. After incubating for 1 h at 37°C, the solution was aspirated and each well was washed with 350 μL of a 1× Wash Solution. The remaining liquid was removed from all wells, and each well was washed three times. Then, 100 μL of Detection Reagent B working solution was added to each well, and the plate was incubated for 30 minutes at 37°C. The aspiration/wash process was repeated 5 times. Next, 90 μL of Substrate Solution was added to each well. The plate was incubated for 15 to 25 minutes at 37°C, and 50 μL of Stop Solution was added to each well to stop the reaction. A microplate reader was immediately used to measure absorbance at 450 nm.

### Immunofluorescence

Cells were washed, fixed in 4% paraformaldehyde, washed twice in PBS, and then permeabilized with 0.2% Triton X-100 for 15 min before blocking with Immunol Staining Fix Solution (Beyotime, Shanghai, China) for 30 min. After incubation with the primary antibody at 4°C overnight (mouse anti-p65), the cells were then washed and incubated with Goat anti-Mouse IgG (H+L) Highly Cross-Adsorbed Secondary Antibody, Alexa Fluor 488 and DAPI (0.1 mg/mL) at 37°C for 1 h. The cells were visualized using a confocal laser scanning microscope(SP-II; Leica Microsystems, Wetzlar, Germany).

### Statistical analysis

All experiments were performed at least in triplicate. Data were presented as the mean±SD, and statistical significances were analyzed by a paired-samples t-test or one-way analysis of variance (ANOVA). The least significant difference method (LSD) was used to compare each of the treatment groups and the control group. P-values less than 0.05 were considered to be statistically significant.

## Results

### 1. The level of UBC9 expression in HCC cell lines, LO2 and the activated LX-2 cells

**Fig 1:**
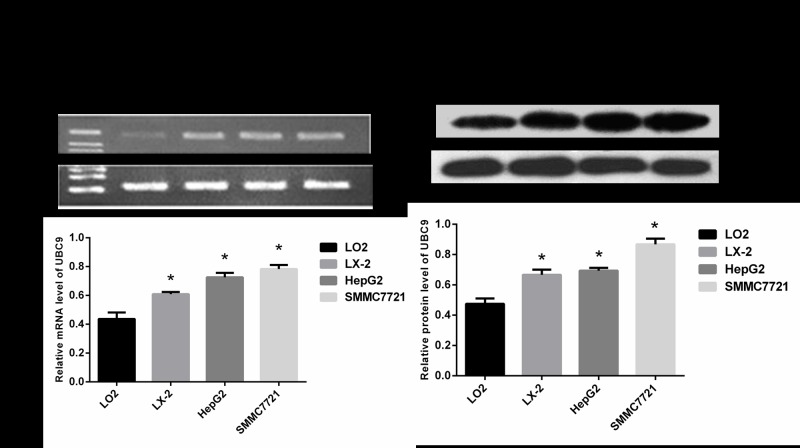
UBC9 expression levels in the normal liver cell line LO2, hepatic stellate cell line LX-2 and hepatoma cell lines HepG2 and SMMC-7721. (A) Top: UBC9 mRNA was examined in LO2, LX-2, HepG2 and SMMC-7721 cells by RT-PCR. Bottom: Graph of relative ratios of UBC9 mRNA to β-actin in each cell line. (B) Top: UBC9 protein expression was examined in LO2, LX-2, HepG2 and SMMC-7721 cells by Western blot. Bottom: Graph of the relative ratio of UBC9 protein to β-actin in each cell line. *P < 0.05 compared with LO2, n = 4.

To observe the expression level of UBC9 in activated LX-2 cells, we employed RT–PCR and Western blot analyses to measure the expression of UBC9 in the normal liver cell line LO2, hepatic stellate cell line LX-2, and HCC cells lines HepG2 and SMMC-7721. The UBC9 levels in activated LX-2 cells, HepG2 and SMMC-7721 were increased compared with LO2, and the expression of UBC9 in activated LX-2 cells HepG2 and SMMC-7721 were no significant differences(Fig 1A and 1B).

### 2. Efficiency of UBC9 induced by UBC9 shRNA in activated LX-2

**Fig 2:**
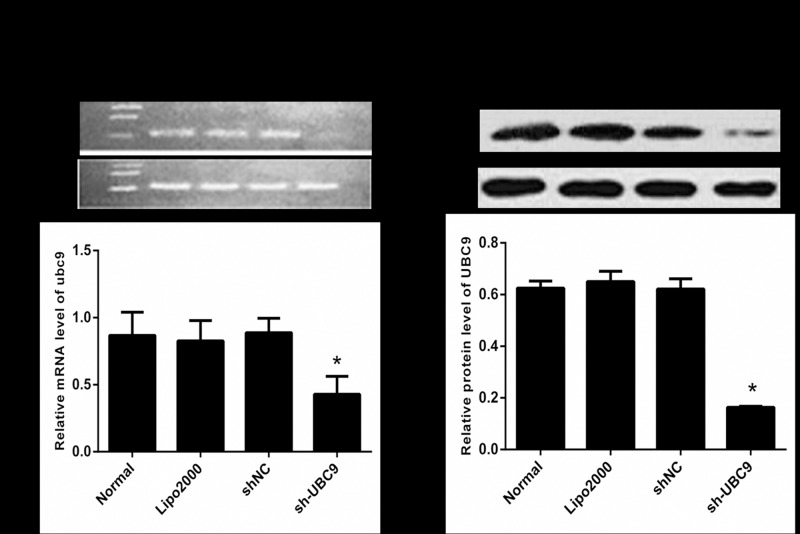
RT-PCR and Western blot analyses of UBC9 expression in activated LX-2 cells. (A) Top: UBC9 mRNA was examined in the Normal, Lipo2000, shNC, and sh-UBC9 groups by RT-PCR. Bottom: Graph of the relative ratios of UBC9 mRNA to β-actin in each group. (B) Top: UBC9 protein expression was examined in the Normal, Lipo2000, shNC, and sh-UBC9 groups by Western blot. Bottom: Graph of the relative ratios of the UBC9 protein to β-actin in each group. *P < 0.01, compared with NC shRNA group, n = 4.

To investigate the effect of UBC9 shRNA transfection on the UBC9 expression level in activated LX-2 cells, the level of UBC9 was measured by RT-PCR and Western blot after transfection. RT-PCR revealed that the UBC9 levels significantly declined in activated LX-2 cells as a result of transfection with UBC9 shRNA. The Western blot results were identical with RT–PCR. These results suggested that UBC9 shRNA could effectively down-regulate UBC9 expression in activated LX-2 cells (Fig 2A and 2B).

### 3. Knockdown of UBC9 inhibited ECM expression in activated LX-2 cells

**Fig 3:**
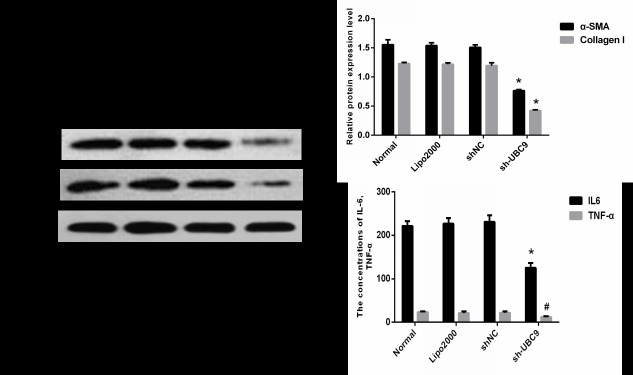
UBC9 knockdown influences ECM expression. (A) Representative images of 3 independent experiments with similar results are presented. Down-regulation of UBC9 reduced α-SMA and Collagen I protein. (B) Graph of the relative ratios of both α-SMA and Collagen I protein to β-actin in each group. *P < 0.05 compared with NC shRNA group, n = 4. (C) IL-6 and TNF-α concentrations in the supernatant of activated LX-2 cells was examined in the Normal, Lipo2000, shNC, and sh-UBC9 groups. ^**#**^P <0.05 and*P < 0.001 compared with NC shRNA group, n = 4.

Activated HSC express α-SMA and Procollagen I protein, which are markers for the activation of HSC. Activated HSCs secrete TNF-α, IL-6, HGF, fibroblast growth factor and other cytokines[6]. To observe whether down-regulation of UBC9 affected the activation of LX-2 cells, activated LX-2 cells were transfected with UBC9 shRNA. Both α-SMA and Collagen I protein expression were reduced in activated LX-2 cells (Fig 3A and 3B). We also found that TNF-α and IL-6 secretion were significantly reduced in transfected cells compared with the control (Fig 3C). These results suggested that down-regulation of UBC9 expression inhibits the activation of LX-2 cells (S1 Table).

### 4. Knockdown of UBC9 inhibited activated LX-2 cell proliferation

**Fig 4:**
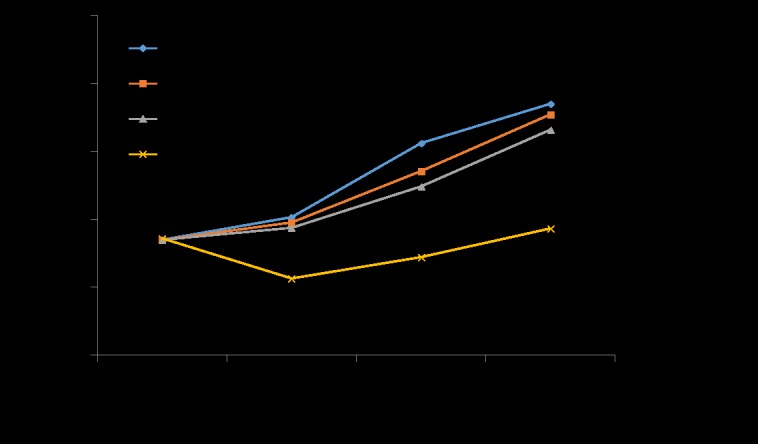
Cell proliferation was assessed using the MTT assay. Activated LX-2 cells expressing UBC9 shRNA exhibited a significant reduction in proliferation compared with NC shRNA groups. *P < 0.05, n = 4.

To study the role of UBC9 in the growth of activated LX-2 cells, UBC9 shRNA was transfected into activated LX-2 cells, and cell growth was detected using an MTT assay. As shown in Fig 4, after transfection, the growth of activated LX-2 cells was significantly inhibited compared with negative control, Lipo2000 and non-treated cells. These results suggested that down-regulation of UBC9 strongly suppresses the growth of activated LX-2 cells.

### 5. The effect of UBC9 on apoptosis-related protein expression in activated LX-2 cells

**Fig 5:**
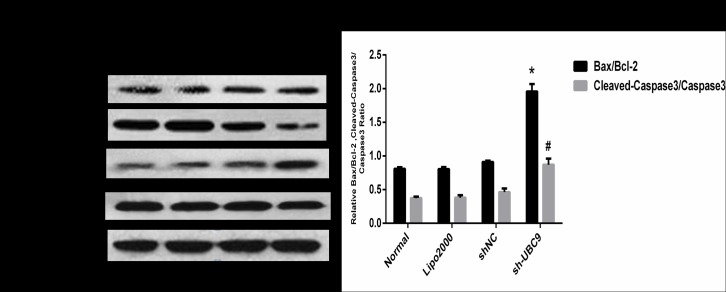
Down-regulation of UBC9 influences the expression of apoptosis-regulated proteins. (A) Bcl-2, Bax, Cleaved-Caspase3 and Caspase3 protein expression was examined in the Normal, Lipo2000, shNC, and sh-UBC9 groups by Western blot. (B) Graph of the relative ratios of Bax/Bcl-2 and Cleaved-Capase3/Capase3 in each group. *P < 0.05 and ^**#**^P <0.001compared with the NC shRNA group, n = 4.

As an anti-apoptotic gene, the main physiological function of Bcl-2 is to inhibit cell apoptosis[18]; however, Bax and Caspase3 promote apoptosis[19]. Therefore, we hypothesized that UBC9 may promote apoptosis in activated LX-2 cells by reducing the expression of Bcl-2. After transfection with UBC9 shRNA, Western blot analysis showed that down-regulation of UBC9 significantly increased the Bax/Bcl-2 and cleaved-capase3/pro-capase3 ratio in activated LX-2 cells (Fig 5A and 5B). These results suggested that down-regulation UBC9 may be a major contributing factor for the high rate of apoptosis in activated LX-2 cells.

### 6. Knockdown of UBC9 promotes activated LX-2 cell apoptosis

**Fig 6:**
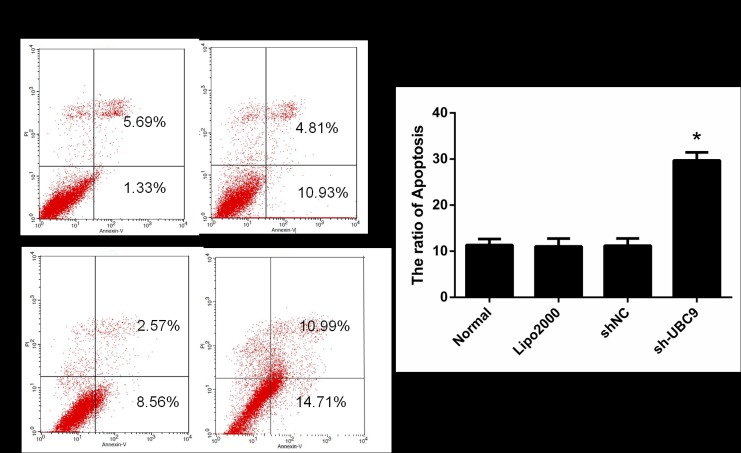
Apoptosis was detected by flow cytometry. The number of apoptotic cells was increased in activated LX-2 cells expressing the indicated shRNA compared with the Normal, Lipo2000 or control group. *P < 0.05 compared with Control shRNA group, n = 4.

To determine the influence of UBC9 shRNA on apoptosis in activated LX-2 cells, apoptosis was assessed by flow cytometry. Compared with cells treated with the negative control, Lipo2000 and non-treated cells, apoptosis was significantly promoted **(**Fig 6A and 6B). (S2 Table)

### 7. Effect on cell cycle progression after transfection with UBC9 shRNA in activated LX-2 cells

**Fig 7:**
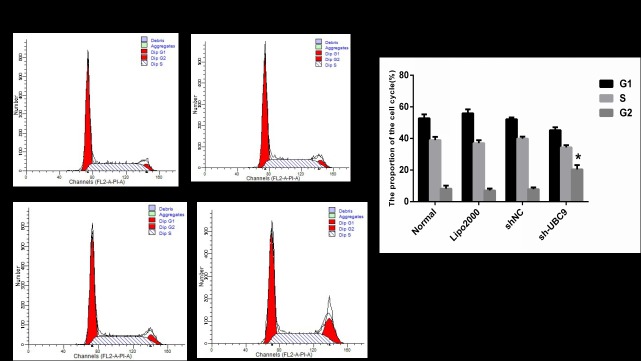
Cell cycle distribution was assessed using the Cell Cycle Analysis Kit. Knockdown of UBC9 exhibited a significantly increase the number of cells in the G2 phase compared with the scrambled control group in activated LX-2 cells. *P < 0.001 compared with the Control shRNA group, n = 4.

To determine the effect on cell cycle progression after transfection with UBC9 shRNA in activated LX-2 cells, cell cycle progression was examined by flow cytometry. Cell cycle analysis revealed that suppression of UBC9 expression caused significant inhibition of cell cycle progression, leading to the selective accumulation of cells in the G2 phase compared with negative control cells (Fig 7A and 7B). (S2 Table)

### 8. Down-regulation of UBC9 expression inhibited the NF-κB signaling pathway in activated LX-2 cells

**Fig 8:**
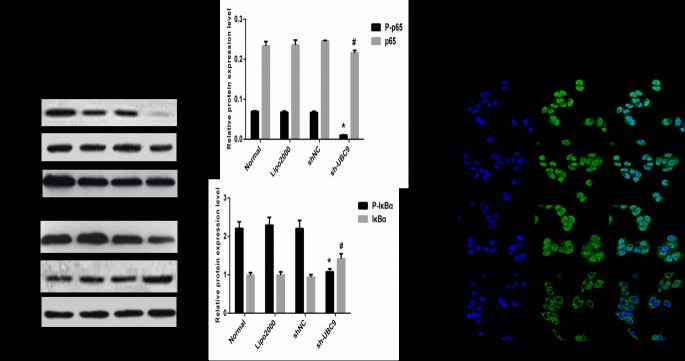
UBC9 knockdown influences the expression of multiple downstream genes. (A) Representative images of 3 independent experiments with similar results are presented. Downregulation of UBC9 expression decreases the expression of the P-p65 and P-IκBα proteins, but increased the expression of the IκBα protein. (B) Graph of the relative ratios of the P-p65, p65, P-IκBα and IκBα proteins to β-actin in each group. *P < 0.01 and ^**#**^P <0.05 compared with NC shRNA group, n = 4.(C) The subcellular localization of p65 in activated LX-2 cells treated with UBC9 shRNA were examined by confocal microscopy analysis with a confocal microscope.

NF-κB plays a broad and important role in the immune response, inflammatory response, cell survival, proliferation, differentiation and apoptosis and exists widely in eukaryotic cells. Activation of the NF-κB signaling pathway is closely related with the phosphorylation and degradation of IκBα[20]. After transfection with UBC9 shRNA, Western blot was utilized to detect protein expression of P-IκBα, IκBα, P-p65 and p65 in activated LX-2 cells. The results showed that downregulation of UBC9 expression significantly decreased the P-p65 and P-IκBα expression levels, and IκBα protein expression was increased in activated LX-2 cells (Fig 8A and 8B). The immunofluorescence assay was performed in our study to further observe the localization of NF-κB in activated LX-2 cells affected by UBC9 expression. Our results showed that UBC9-shRNA treatment significantly decreasing phosphorylation of p65 by inhibited the ranslocation of ReIA/p65 from cytoplasm to nucleus(Fig 8C). These results suggested that down-regulation of UBC9 expression could inhibit the NF-κB signaling pathway in activated LX-2 cells.

## Discussion

In this study, we demonstrated that UBC9 expression was significantly up-regulated in activated LX-2 cells. Knockdown of UBC9 significantly inhibited HSC proliferation and reduced the expression levels of a-SMA and collagen I. Downregulation of UBC9 expression in activated LX2 also caused cell cycle arrest at the G2 phase and induced apoptosis in HSCs mediated by the up-regulation of the protein Bax/Bcl-2 and Cleaved-Capase3/Capase3 ratio. Furthermore, knockdown of UBC9 prevented liver fibrosis by inhibiting HSC proliferation and inducing HSC apoptosis by inhibiting the NF-κB signaling pathway.

UBC9 has been identified as a novel regulator of fibrosis. Scholars have demonstrated that inhibition of SUMOylation by knockdown of UBC9 almost completely prevented the development of fibrosis and inhibited the canonical TGF-β/Smad signaling pathway in experimental models[13]. In this study, we demonstrate that UBC9 expression in LX-2, HepG2, and SMMC-7721 was significantly increased compared with LO2, suggesting that UBC9 might play a role in of liver fibrosis. Furthermore, UBC9 might promote hepatic fibrosis through activation of HSCs.

First, after down-regulation of UBC9, the expression levels of markers of HSC activation, including a-SMA and Collagen I, were reduced in activated LX-2 cells. In addition, TNF-a and IL-6 secretion by activated LX-2 cells was also reduced. In the liver, TNF-a, which plays a significant role in necrosis and apoptosis, tissue damage, inflammation and fibrosis, is mainly secreted by Kupffer cells, monocytes, macrophages, HSCs and other cells[21]. TNF-a is involved in the regulation of hepatic fibrosis through a variety of mechanisms. TNF-a promotes fibroblast proliferation, collagen synthesis and transformation into myofibroblasts. TNF-α also increases liver fibrosis by promoting activated HSCs to produce a large number of ECM proteins and to secrete several soluble cytokines, such as TGF-β, IL-1, IL-6, VEGF and ET-1[22]. IL-6 is a multifunctional cytokine that has both pro- and anti-inflammatory properties[23]. Additionally, IL-6 is a key player in the network of inflammatory mediators and plays an important role in the inflammatory response. Increased IL-6 expression in vivo can lead to several diseases, including rheumatoid arthritis, glomerulonephritis, Crohn’s disease (CD) and Castleman’s disease. One study found that patients with chronic kidney disease exhibited increased expression of IL-6[24]. Furthermore, IL-6 induces the production of acute phase proteins, such as hepatocyte stimulating factor (HSF) [25].

Stimulating apoptosis in HSCs is considered to be a necessary step to resolve liver fibrosis[17,26,27]. Thus, elucidation of the mechanisms involved in HSC apoptosis and identification of the key players in this process can lead to the discovery of new anti-fibrotic targets and the development of novel therapeutic strategies. In combination with the flow cytometric analysis results, we suggest that knockdown of UBC9 promotes cell apoptosis in activated LX-2 cells, and the pro-apoptosis effect of knockdown UBC9 on activated LX-2 cells was further confirmed by the increase of the Bax/Bcl-2 and cleaved-Caspase3/Caspase3 ratio.

The NF-κB signaling pathway plays an active role in a number of chronic liver diseases[16,28]. Evidence suggests that the activity of NF-κB is increased in activated HSCs[29,30] and that inhibition of HSC apoptosis promotes liver fibrosis[30,31]. Inhibition of the NF-κB signaling pathway is typically associated with the induction of apoptosis in activated HSCs and reversal of experimentally induced liver fibrosis[30]. For example, NF-κB proteins play a major role in factor decoy, which affects CCl4-induced liver injury and fibrosis by activated HSCs and inhibition of HSC apoptosis [32]. SUMOylation of important protein factors in the NF-κB signaling pathway, such as IκBα and NEMO, could lead to a modified response. IκBα was the first protein in the NF-κB signaling pathway discovered to be SUMO-modified[20]. Scholars have also demonstrated UBC9 silencing reduced the capture of IkBa modified with SUMO-ubiquitin hybrid chains that display a defective proteasome-mediated degradation. Thus, silencing of UBC9 leads to loss of phosphorylation of IkBa, attenuation of SUMO-2-Ubiquitin heterologous chains on IkBa, decreased proteasomal degradation of IkBa and a delay in NF-kB activation[33]. So in our study, UBC9-shRNA treatment significantly reduced the phosphorylation of p65 and IκBα, but increased the expression of IκBα. Our results also showed that UBC9-shRNA treatment significantly decreasing phosphorylation of p65 by inhibited the ranslocation of ReIA/p65 from cytoplasm to nucleus. These all suggests that down-regulation of UBC9 expression negatively controls NF-κB signaling pathway.

In summary, our findings suggest that UBC9 plays a critical role during hepatic stellate cell activation and apoptosis by inhibiting the NF-κB signaling pathway. Down-regulation of UBC9 expression induced LX-2 cell apoptosis and prompted the cell to return to a quiescent state, which may have therapeutic potential for the treatment of liver fibrosis. To the best of our knowledge, this is the first report of UBC9 down-regulation during liver fibrosis *in vitro* and is also the first report of UBC9 function in organic fibrosis reversion. Although future studies should be performed to confirm the effects, UBC9 potentially serves as an ideal target for the prevention and treatment of liver fibrosis.

## Supporting information

S1 Table Secretions of TNF-α and IL-6 by LX-2 cells transfected with UBC9 shRNA. (DOCX)

S2 Table Effect on cell cycle progression and apoptosis after transfection with UBC9 shRNA in activated LX-2 cells. (DOCX)
