# Supplementary material for: Downregulation of UBC9 promotes apoptosis of activated human LX-2 hepatic stellate cells by suppressing the canonical NF-κB signaling pathway
Source: PLoS One. 2017 Mar 30;12(3):e0174374. doi: 10.1371/journal.pone.0174374 (PMC5373541; doi:10.1371/journal.pone.0174374)
Supplement: S1 Table — (DOCX) [file pone.0174374.s001.docx]

**Table1A Secretions of TNF-α by LX-2 cells transfected with UBC9 shRNA.**

The concentrations of TNF-α in the supernatant of LX-2 cells was examined in the Normal, Lipo2000, shNC, and sh-UBC9 groups.*P < 0.001, compared with NC shRNA group, n = 4.

**Table1B Secretions of IL-6 by LX-2 cells transfected with UBC9 shRNA.**

The concentrations of IL-6 in the supernatant of LX-2 cells was examined in the Normal, Lipo2000, shNC, and sh-UBC9 groups. *P < 0.001, compared with NC shRNA group, n = 4.
