# Supplementary material for: Downregulation of UBC9 promotes apoptosis of activated human LX-2 hepatic stellate cells by suppressing the canonical NF-κB signaling pathway
Source: PLoS One. 2017 Mar 30;12(3):e0174374. doi: 10.1371/journal.pone.0174374 (PMC5373541; doi:10.1371/journal.pone.0174374)
Supplement: S2 Table — (DOCX) [file pone.0174374.s002.docx]

**Table2A Apoptosis was detected by flow cytometry.**

The number of apoptotic cells was increased in activated LX-2 cells expressing the indicated shRNA compared with the Normal, Lipo2000 or control group. *P < 0.05 compared with Control shRNA group, n = 4.

**Table2B Cell cycle distribution was assessed using the Cell Cycle Analysis Kit.**

Knockdown of UBC9 exhibited a significantly increase the number of cells in the G2 phase compared with the scrambled control group in activated LX-2 cells. *P < 0.001 compared with the Control shRNA group, n = 4.
